# Patient reported outcome measures in rare diseases: a narrative review

**DOI:** 10.1186/s13023-018-0810-x

**Published:** 2018-04-23

**Authors:** Anita Slade, Fatima Isa, Derek Kyte, Tanya Pankhurst, Larissa Kerecuk, James Ferguson, Graham Lipkin, Melanie Calvert

**Affiliations:** 1 Centre for Patient Reported Outcome Research, College of Medical and Dental Sciences, University of Birmingham Edgbaston, Birmingham, B15 2TT UK; 2 Institute of Applied Health Research, University of Birmingham, Birmingham, UK; 3 University Hospital Birmingham, Birmingham, UK; 4 Birmingham Childrens and Womens Hospital, Birmingham NHS Foundation Trust, Birmingham, UK; 5 Birmingham Health Partners, Birmingham, UK; 6 Public Health England, Birmingham, UK

**Keywords:** Rare diseases, Rare disorders, Patient reported outcome measures, Outcome assessments, Quality of life

## Abstract

**Background:**

Rare diseases can lead to a significant reduction in quality of life for patients and their families. Ensuring the patients voice is central to clinical decision making is key to delivering, evaluating and understanding the efficacy of therapeutic interventions. Patient reported outcome measures (PROMs) are used to capture the patient’s views about their health status and facilitate our understanding of the impact of these diseases and their treatments on patient’s quality of life and symptoms.

**Main text:**

This review explores some of the current issues around the utilisation of PROMs in rare diseases, including small patient populations and dearth of valid PROMs. Difficulties in validating new or current PROMs for use in clinical trials and research are discussed. The review highlights potential solutions for some of the issues outlined in the review and the implementation of PROMs in research and clinical practice are discussed.

**Conclusion:**

Patient input throughout the development of PROMs including qualitative research is essential to ensure that outcomes that matter to people living with rare disease are appropriately captured. Given the large number of rare diseases, small numbers of patients living with each condition and the cost of instrument development, creative and pragmatic solutions to PROM development and use may be necessary. Solutions include qualitative interviews, modern psychometrics and resources such as item banking and computer adaptive testing. Use of PROMs in rare disease research and clinical practice offers the potential to improve patient care and clinical outcomes.

## Background

There are approximately 7000 identified distinct rare diseases affecting around 350 million people worldwide [1]. In the European Union, a disease is deemed to be rare when it affects less than 1 per 2000 persons [2]. In the United States, a disease is defined as rare when it affects fewer than 200,000 people at any given time. An estimated 80% of rare diseases have a genetic origin and the remaining 20% are as a result of infections (bacterial or viral), environmental factors, allergies, proliferative and degenerative causes [2]. Approximately 75% of rare diseases affect children, 30% of whom die before their 5th birthday [3]. Despite this, approximately 95% of rare diseases have no Food and Drug Administration (FDA) approved treatment. While only 140 orphan medicines have been authorised for use in the European Union since 2001, and 60% of these were designated for use in paediatric conditions [1, 4].

### The challenges faced by patients with rare disease

The impact of rare diseases can be diverse and include loss of physical function, cognitive and communication impairments, as well as emotional problems and social isolation. This may present challenges for patients in their social interactions, work, and education and can also have an adverse impact on the quality of life of other family members [5].

Many patients with a rare disease find themselves with unmet clinical needs due to a number of issues associated with access to clinical care and information [6, 7]. Patients with the same diagnosis may present differently because of genetic variations and timing of presentations. This can result in a delay to diagnosis, or misdiagnosis, because they cannot access specialists with knowledge of their disease [7, 8]. Even after receiving a diagnosis, accessing therapeutic interventions and appropriate drugs may be difficult as treatment options maybe non existent or restricted and often costly [8, 9]. Specialist clinics are often held infrequently, are limited in number and are located in regional centres, therefore patients have to travel considerable distances to access expert care [7, 9]. This can result in significant financial burdens associated with travel costs and the need to take time off work to undergo therapeutic procedures or clinic visits and may prevent some patients from accessing the expert care they need [8, 9]. Accessing quality information can also be difficult for both patients and their families because little may be known or published about their rare disease, therefore limiting their access to healthcare options or informed care [7, 10].

The impact of rare diseases on a patient’s daily activities means individuals are dependent on families or carers to manage their everyday needs [3]. This loss of autonomy can have a negative impact on their quality of life (QoL) and has implications for their family and carers, given the burden of care and the young age of many of those diagnosed [2]. As a result, families have reported psychosocial concerns and feelings of isolation and depression [11]. The diverse presentations and progressive nature of many rare diseases make it important to chronicle an individual’s natural history and the extent to which their health and social care needs are changing [8].

### What are patient reported outcome measures?

One way of monitoring changes in the natural history and disease progression of a rare disease is through the use of patient reported outcome measures (PROMs) [12, 13]. PROMs are reports directly obtained from patients about their health status/condition or treatment without interpretation by a clinician [14, 15]. PROMs are used in research and clinical settings to provide the patient’s views about a range of issues including symptom severity, function, psychological problems, treatment satisfaction and health-related quality of life (HRQoL) [16].

PROMs are generally characterized as generic or disease-specific measures. Generic measures are designed to assess any disease condition or general population sample. These measures can be used to compare the health status across and between different disease groups [12, 17]. However, generic measures may exclude important aspects that are relevant to specific groups [18]. Condition-specific measures are designed to assess the severity of symptoms, or functional limitations specific to a particular health condition or diagnostic group. Condition-specific PROMs may be more sensitive when monitoring symptoms and detecting changes in health within a specific homogeneous group [19].

It is also recognised that for a condition specific PROM to have content validity it should have been developed with input from service users who have the lived experience of the condition [19, 20]. The European Medical Agency (EMA) [21] and the Food and Drugs Agency (FDA) have recognised the importance of including the patient’s perspective in development of PROMS and the FDA guidelines state that patient input is a requirement for applications using PROMs to support medical labelling claims [15, 22]. It has also been demonstrated that PROMs developed using the patient’s perspective are more robust and provide more sensitive and specific measurements [19].

### PROMs available for use in patients with rare diseases

PROMs data, if collected, analysed and reported appropriately, can inform shared-decision making, pharmaceutical labelling claims, clinical guideline development and health policy [23]. PROMs also have an important role as primary or secondary endpoints in clinical trials and other epidemiological studies, developing and evaluating new therapies [24]. However, there are a number of issues relating to the use of PROMs in rare diseases. These include: a dearth of PROMs validated for the target population, especially for children and adolescents; sampling issues, data collection and statistical analysis issues secondary to small sample sizes and heterogenous study populations; and diverse contexts for recruitment such as multi-centre international studies.

### PROMs in rare disease research

Despite these issues, however, PROMs are being used to assess the impact of rare diseases on quality of life. A systematic review on quality of life in rare genetic conditions concluded that the Short Form-36 item health survey (SF-36), Cystic fibrosis questionnaire and Dermatologic-specific QoL scales were the most frequently used measures in primary studies [25]. Another review focussing on children and adolescents with rare diseases, identified that the Child Health and Illness Profile (CHIP), pediatric quality of life inventory (PedsQL) [26] and KIDSCREEN [27] questionnaires were the measures of choice for measuring HRQoL [25].

As well as these generic HRQoL tools, there are a number of PROMs that have been developed specifically for rare diseases, these include: the phenylketonuria-specific Quality-of-Life questionnaire (PKU-QoL) [28, 29]; Birdshot chorioretinopathy (BCR) Birdshot Disease & Medication Symptoms Questionnaire (BD & MSQ), quality of life impact of BCR (QoL-BCR) and the QoL impact of BCR medication (Qol-Meds) [30]; the Kids’ ITP tool used for children with immune thrombocytopenic purpura (ITP) [31]; and the Fabry-specific Paediatric Health and Pain Questionnaire (FPHPQ) [32].

Despite these examples, most rare diseases lack disease-specific PROMs that can be used to gain a better understanding of the issues that patients experience [33]. A systematic review focussing on the impact of enzyme replacement therapy on HRQoL in Fabry disease (FD) found HRQoL was lower in the FD population and efficacy of current therapies was inconclusive [34]. They found limitations in some of the generic HRQoL PROMs used in the study, and suggested that a FD specific PROM would be more accurate when monitoring patients.

### Why PROMs are important in clinical practice

Routine use of PROMs in clinical practice has been shown to improve patient satisfaction with their care, symptom management, quality of life and survival rates [35–37]. It may also benefit clincians by improving clinician satisfaction, helping reduce burnout and increasing workflow efficiency, as routine screening questions may be asked in the waiting room and clinicians can use results from PROMs to inform the consultation [14]. This can help promote patient-centred care by improving communication between clinicians and patients about their disease progression and the impact of prescribed treatments [38]. PROMS can also support clinical decision-making when prescribing therapeutic interventions and managing side effects. In some specialities (e.g. cancer, mental health), PROMs are used to assess disease severity, monitor symptoms and responses to therapeutic interventions [39]. PROMs can also identify adverse events and screen patients for physical and emotional problems requiring additional clinical intervention [40, 41]. While these are considerable advantages, PROMs data is still not routinely collected in all areas of clinical practice especially in the case of rare diseases [12].

### Understanding what matters to patients living with rare diseases

In order for PROMs to be effective in clinical practice they have to capture the disease characteristics that matter to the patient. Selection of suitable PROMs should reflect these properties and domains as well as reflecting the natural disease history and prognosis. This can be challenging because of the heterogeneity of patients experiences, and differences in presentations caused by cultural influences, genetic and phenotype variations [42]. Therefore its important to understand these variances when establishing key characteristics and selecting suitable PROMs. A recent taskforce report suggested one way of identifying patient preferences in rare diseases was through the use of qualitative interviews with patients, carers and their families [12]. These could be used to elicite common core domains and symptoms. They also suggested key areas for research focus should include:

1. Research priorities
2. Outcomes that matter to patients (including PROMs)
3. The views of patient and clinicians regarding the use of PROMs in research and routine clinical practice.

Addressing these key areas would ensure patients voices are central to research and clinical practice. This would enhance our understanding of the experiences of people living with a rare disease, and help ensure that outcomes that matter are encapsulated in research and clinical practice. Patient Centred Outcome Measures (PCOMs) as proposed by Morel and Cano [43] can also provide additional benefits to health care providers by addressing the important issues that matter to patients, whilst providing a measure of patient benefit.

In addition to qualitative interviews other evidence to inform PROM development may include: literature reviews; media access, genotype-phenotype databases; observational studies; case histories; rare disease registries; and observer information e.g. parents and carers [12]. Access to patient forums and blogs may also help researchers to gain insights into the issues that matter to patients [44]. Patient and public involvement (PPI), and patient advocacy groups also present additional opportunities for accessing patients’ viewpoints [45, 46]. Encapsulating patients viewpoints into PROMs and collaborations between industry, academics and patient partners can improve the quality of data collected [47–49]. As demonstrated by Mesa et al. [48] who identified which symptoms were important to patients with myelofibrosis (a rare myeloproliferative neoplasm) through qualitative interviews. They used this information to create and test an appropriate PROM for use in a clinical trial of ruxolitinib [50]. Using a PROM that was meaningful to the patients resulted in more than 95% compliance in the trial and the results demonstrated that the experimental group had a 50% or greater improvement in comparison to the control group [47]. This resulted in approval of ruxolitinib by the FDA and EMA [47].

### Using PROMs in patient registries

Patient registries are also a useful tool for the development of rare disease research and can help improve patient care and services, as well as development of new treatments [2, 51]. Rare disease registries have often grown organically and are usually initiated by organisations including patients, family, carers and advocacy groups, as well as industry partners, health services and clinicians [52]. Registries are important resources for information on the natural history of diseases, supporting enrollment of patients to research and to assess the impact of new therapies in real-world settings [51]. PROMs are increasingly being used in patient registries to understand the patient perspective and disease management strategies [53].Incorporation of PROMs in registries can improve our understanding of the impact of symptoms and QoL over the course of the disease and treatment [52]. There are a large number of registries for rare diseases and and a number of them are collecting PROM data [54]. Examples of these registries can be found online, including the UK Cystic Fibrosis registry [55] and the Cystic Fibrosis Foundation United States (US) [56]. The lysosomal storage disorder registry is also an example of how conditions with a common link can be accomodated together e.g. Fabry disease, [57] Pompe [58] and Gaucher [59]. This approach allows common themes to be identified and pools resources for families, clinicians and researchers.

### Proms for assessing service delivery

As well as being an important tool in clinical care PROMs can be used to evaluate service delivery as demonstrated by the use of the EQ-5D for audit/benchmarking purposes in the UK National Health Service (NHS) since 2009. The EQ-5D in conjunction with disease specific measures are used to measure HRQoL change pre and post-surgery in Hip and knee replacements, hernia repairs and varicose vein surgery [60, 61]. PROM data collected at a national level in this way can inform patients’ decisions regarding the selection of their preferred health care provider. However such an approach is not without challenges, for example reducing levels of missing data and providing information on PROM results to patients in an easily accessible way can be difficult to achieve [62, 63]. Using PROMs as part of routine care would give patients an important say in their treatment and could be used as a performance metric to compare the quality and effectiveness of care providers. This is especially important given the complexities associated with their care pathways and dearth of specialist services [64].

The diversity of PROMs currently used in clinical trials and practice makes it difficult to compare results when different outcomes have been utilised. Consequently, there have been moves to develop recommended core outcome measures for different conditions for use in clinical trials and service evaluation. This approach has been developed by the International Consortium for Health Outcomes Measurement (ICHOM), their remit is to incorporate patient and clinician view points and develop standardised sets of outcome measures for specific medical conditions based on concerns that matter to patients, these can then be used to evaluate and compare services [65]. Other approaches to developing core outcome sets include those developed by Core Outcome Measures in Effectiveness Trials (COMET) which brings together experts and researchers in specific conditions to develop core outcome sets recommended for use in trials and clinical practice. Another group taking a similar route includes the Outcome Measures in Rheumatology (OMERACT) initiative which also aims to develop a consensus on which outcome measures should be used in autoimmune and musculoskeletal conditions [66].

### Selecting reliable and valid PROMS

Having identified which issues matter most to patients it then becomes important to ensure that selection of potential PROMs reflects those issues. Additional considerations include the psychometric validity of the PROM and the context in which PROM data are being collected. Ensuring the reliability, validity and acceptability of the measure is a crucial component of selecting PROMs for use in rare diseases. Evaluating the measurement properties of PROMs in rare disease is challenging because of the small numbers of patients and heterogenous populations. Therefore, some of the usual options for choosing, adapting or developing PROMs are not always appropriate or available for this group of patients and may require adaptation [12]. Key to this process is understanding the construct of interest (COI) and a clearly defined rationale for using PROMs within a research study in order to reach a meaningful conclusion about the outcome/endpoints that will be measured [67]. When deciding on the PROM to use, the COI should meet the aim of the research study e.g. symptom burden, or HRQoL [67]. Additionally, the measure should be selected according to the population of interest e.g. disease characteristics. Information gleaned from qualitative interviews on which symptoms and outcomes are important to the patients can inform this process. However, given the challenges of recruitment in rare diseases it may be more pragmatic to use existing measures with additional items to capture specific disease issues or domains [12].

A systematic review may be required to identify the most suitable PROMs [67]. Guidelines developed by the COnsensus-based Standards for the selection of health Measurement INstruments (COSMIN) initiative aims to help improve the selection of outcome measurement for research and clinical practice [68]. This has included the development of a critical appraisal tool for evaluating the methodological quality of studies evaluating health measures such as PROMs. Systematic reviews can be used as tools to identify gaps in knowledge on the quality/measurement properties of PROMs or potential PROMs for use in rare diseases [69]. An example of how COSMIN guidelines can be used in practice is demonstrated in a systematic review of PROMs used in Primary Sclerosing Cholangitis (PSC), conducted by the authors. The review found that a wide diversity of PROMs were used to measure HRQoL and symptom burden in patients with PSC. However, the quality of the evidence to support reliability and validity in the most commonly used measures was generally poor and only one had been validated within this population following FDA guidelines [70, 71].

Additional sources of information include databases such as ePROVIDE™ which has a range of information on PROMs and includes some critical review on the measurement properties for each PROM [72]. Additional databases include the Patient-Reported Outcomes Measurement Information System (PROMIS) which is a co-operative group program of research which aims to develop, validate and standardise item banks to capture PROM data across a wide range of conditions and domains for use in a range of chronic disorders and conditions [73].

PROMIS uses item response theory (IRT) and computer adaptive testing (CAT) technology to generate and validate item banks for specific domains [74]. Access to electronic forms of data collection using different psychometric models offer some potential solutions to the issues that arise from using traditional methods such as classical test theory (CTT). These models include IRT, and Rasch measurement theory (RMT). Increasingly these different methods are being used to modify or evaluate PROMs for use in rare diseases [43, 75].

While it is important to collect a comprehensive range of information using PROMs often data includes a wide range of domains. Collecting information on every PROM for every domain can be burdensome and result in PROM fatigue and missing data. CAT technology is effective in reducing this burden, by only presenting a small but specific number of questions, sequentially selected based on responses to previous questions [74]. This approach also means that condition specific items as highlighted by the patients living with the rare disease can be incorporated with readily available tools [76]. This allows tailoring of the PROM to a patient’s specific symptoms or needs without the need to develop a completely new instrument. One of the drawbacks of this method are the large sample sizes required to carry out psychometric analysis for validation of the PROMs [77]. Using RMT is a potential solution to this issue. Rare group populations can be combined with populations with similar disease presentations [78, 79]. Examination of differential item functioning (DIF) analysis by diagnostic group could identify if responses from these groups are equivocal, if they are then both samples can be used to validate PROMs [80]. Items where DIF is an issue can be split and used with the appropriate population in an item bank.

Mattera et al. [76] used concept elicitation and cognitive interviews to refine potential items from the PROMIS physical function (PF) item bank for use with patients with Fibrodysplasia Ossificans Progressiva (FOP) (an ultra-rare and progressive disease). Concept elicitation interviews were used to identify the impact of FOP on daily life and the concepts that mattered to the participants living with FOP. Themes and concepts from the interviews were identified and items that reflected these were established from the selected PROMIS PF items. Cognitive interviews were used to ensure that selected items were relevant and understood by the participants. The findings of the study demonstrated that concept elicitation and cognitive interviews could support development or modification of instruments for use with rare diseases [76].

### Cultural adaptation of PROMS

While pooling data maybe one option, participants recruited for clinical trials might not only be recruited from multiple-centres but also different countries [75]. Therefore PROMs will be required in different languages in order to accommodate this type of rescruitiment. This raises a number of issues. Firstly, PROMs should have been translated using industry standard methods, as both language and cultural adaptations are required [12]. Cultural adaptation ensures that the context and content is appropriate for the population. This aspect of translation is often overlooked when translating PROMs and this can have a negative impact on attrition rates and levels of missing data. There are a number of recommendations for adapting PROMs to ensure cross cultural validity and equivalence [81–83]. Pooling data from international studies maybe the only way of recruiting sufficient sample sizes to test the psychometric properties of a PROM or to establish suitable therapeutic interventions. However, because of the influences of cultural adaptations it then becomes important to ensure the cross-cultural validity and equivalence of PROMs before pooling data. One approach to doing this could be through the use of RMT. By using RMT it is possible to establish whether DIF by country or language is an issue [84]. If DIF by country is not identified then cross-cultural equivalence can be assumed and pooled data results can be utilised in clinical trials [84]. Regnault and Herdman (2015) also suggest using factor analysis and the Universalist model to establish whether the theoretical model fits the data. Confirmatory factor analysis and qualitative interviews are used to evaluate conceptual and cross cultural equivalence of PROMs [83]. Utilising psychometric methods alongside qualitative methods can facilitate identification of the most appropriate PROMs for use in rare diseases and conditions of interest [85].

### Technological advances and prom data collection

The rapid progression of alternative technologies, increased connectivity and ease of administration has seen the increasing use of electronic PROMs (ePROMs), allowing data to be collected internationally, locally and in real time. It also gives patients the freedom to complete PROMs using an electronic device of their choosing e.g. tablets, smartphones etc. and at a time that is convenient for them [86]. This is particularly important for patients with unstable conditions or high symptom burden who are likely to deteriorate rapidly [86]. Patients can also opt to complete PROMs prior to clinical visits [36]. Technological advances also offer alternative and adaptive methods for data collection such as audio options for people with low vision and touch screens that eliminate the need for computer skills [36]. Wearable technology and Telehealth are used to capture clinical data, allowing patients remote access to expert clinical care without the need for expensive travel. Incorporating PROM data capture in addition to clinical data would allow clinicians to evaluate the health status of patients between clinic appointments and monitor the impact or side effects of drug from therapeutic interventions [87, 88].

PROMs can be integrated into electronic healthcare records and the use of predetermined threshold absolute and change scores can generate an automatic notification that can be provided both to the patient and the clinical team where follow-up is required [36]. Appointments can be changed if patients appear to be deterioriating or postponed if patients appear stable. Thereby improving access to timely health care interventions while reducing patients’ health and financial burdens, reducing service costs by utilising health service resources appropriately [35]. The Patient-Centred Outcomes Research Institute (PCORI) have developed a comprehensive guide on how to integrate ePROMS into electronic health records [89].

This paper has highlighted some of the issues that have been identified in using PROMs in rare diseases, however, they are not insurmountable and we have offered a number of potential solutions. Identifying the issues for people living with rare diseases and ensuring that their views are central to health care and policy is paramount. It is also important to remember that having an appropriate PROM in place does not necessarily mean that data can be collected from all patients. Physical limitations, cognitive impairments and age may prevent some patients from completing the PROM. In these instances, alternate methods such as interviews or observer collected data maybe required to enable completion. The International Society for Pharmoeconomics and Outcomes Research (ISPOR) has recently published comprehensive guidance on how this can be achieved [12]. There is also an increasing emphasis on integrating quality PROM data into clinical care enabling dialogue between the clinician and patients and facilitating joint decision making around care options. In some respects this is more important for patients with rare diseases, than other long-term conditions as often their voices are in the minority and their clinical options limited. The use of PROMs potentially provides a more complete picture of their experiences and enables better communication with the clinical team.

## Conclusion

Rare diseases can be chronically debilitating or life-threatening conditions, leading to a significant reduction in QoL for both the patient and their families. Small patient populations and a relative scarcity of medical knowledge and specialists in rare diseases make it challenging to understand the burden of these diseases in patients. The importance of ensuring that the patients voice is central to clinical decision making is key to delivering, evaluating and understanding therapeutic interventions. However, this requires ways of evaluating outcomes that reflect patients needs and concerns as well as clinically driven measures and outcomes. The dearth of valid PROMs for use in many rare conditions and difficulties in validating currently available PROMs makes evaluating effective treatments problematic. Patient input throughout the development of PROMs including qualitative research is essential to ensure that outcomes that matter to people living with rare disease are appropriately captured. Given the large number of rare diseases, small numbers of patients living with each condition and the cost of instrument development, creative and pragmatic solutions to PROM development and use may be necessary, such as the development of item banks and use of CAT. Use of PROMs in rare disease research and clinical practice offers the potential to improve patient care and outcomes.

## Abbreviations

BCR: Birdshot chorioretinopathy
BD&MSQ: Birdshot disease & medication symptoms questionnaire
CAT: Computer adaptive testing
CHIP: Child health and illness profile
COI: Concept of interest
COMET: Core outcome measures in effectiveness trials
COSMIN: Consensus-based standards for the selection of health measurement instruments
CTT: Classical test theory
DIF: Differential item functioning
EMA: European medicines agency
ePROMS: Electronic PROMs
EQ-5D: EuroQoL-5 dimensions
FD: Fabry disease
FDA: Food and drug administration
FOP: Fibrodysplasia ossificans progressiva
FPHPQ: Fabry-specific paediatric health and pain questionnaire
HRQoL: Health-related quality of life
ICHOM: International consortium for health outcomes measurement
IRT: Item response theory
ISOQOL: International society of quality of life research
ISPOR: International society for pharmoeconomics and outcomes research
ITP: Immune thrombocytopenic purpura
LSDs: Lysosomal storage diseases
NHS: National health service
NIH: National institutes of health
PCOMs: Patient centred outcome measures
PCORI: patient-centred outcomes research institute
PedsQL: Paediatric quality of life inventory
PKU-QoL: Phenylketonuria - quality-of-life questionnaire
PPI: Patient and public involvement
PROMIS: Patient-reported outcomes measurement information system
PROMs: Patient reported outcome measures
PSC: Primary sclerosing cholangitis
QoL: Quality of life
QoL-BCR: Quality of life impact of BCR
Qol-Meds: The QoL impact of BCR medication
RMT: Rasch measurement theory
SF-36: 36-item short form health survey
UK: United Kingdom
US: United States
